# A Low-Cost, Computer-Interfaced Drawing Pad for fMRI Studies of Dysgraphia and Dyslexia

**DOI:** 10.3390/s130405099

**Published:** 2013-04-17

**Authors:** Frederick Reitz, Todd Richards, Kelvin Wu, Peter Boord, Mary Askren, Thomas Lewis, Virginia Berninger

**Affiliations:** 1 Instrument Development Lab, Center on Human Development and Disability, University of Washington, Seattle, WA 98195, USA; E-Mails: wuanzinger@gmail.com (K.W.); tnlewis@uw.edu (T.L.); 2 Integrated Brain Imaging Center, Department of Radiology, University of Washington, Seattle, WA 98195, USA; E-Mails: toddr@uw.edu (T.R.); pboord@uw.edu (P.B.); askren@uw.edu (M.A.); vwb@uw.edu (V.B.)

**Keywords:** fMRI, MRI-compatible, dygraphia, dyslexia, lexical, handwriting, LabVIEW

## Abstract

We have developed a pen and writing tablet for use by subjects during fMRI scanning. The pen consists of two jacketed, multi-mode optical fibers routed to the tip of a hollowed-out ball-point pen. The pen has been further modified by addition of a plastic plate to maintain a perpendicular pen-tablet orientation. The tablet is simply a non-metallic frame holding a paper print of continuously varying color gradients. The optical fibers are routed out of the MRI bore to a light-tight box in an adjacent control room. Within the box, light from a high intensity LED is coupled into one of the fibers, while the other fiber abuts a color sensor. Light from the LED exits the pen tip, illuminating a small spot on the tablet, and the resulting reflected light is routed to the color sensor. Given a lookup table of position for each color on the tablet, the coordinates of the pen on the tablet may be displayed and digitized in real-time. While simple and inexpensive, the system achieves sufficient resolution to grade writing tasks testing dysgraphic and dyslexic phenomena.

## 1. Introduction

A functioning writing and reading brain requires a system of language-related neural components to be well-connected and integrated. The long term goal of this project is to understand the neural substrates responsible for the writing/reading brain in children with learning disability. As part of this project, we developed a device for recording handwriting during an fMRI task in children with dysgraphia and dyslexia so that behavior and brain function can be assessed in the same writing-task session.

The MRI environment presents special challenges to sensor design, though many such sensors have been designed, containing only small amounts of metal [1–3], or even circuitry [4]; the magnetic fields in operation within an operating MRI are strong, but finite, such that suitably small device profiles remain acceptable. Zakzanis *et al.* [5] custom-built an fMRI-compatible writing device for investigation of the cerebral correlates of a neuropsychological assessment called the Trail Making Test. With this fiber-optic device, called the “virtual stylus”, they demonstrated fMRI activation in the frontal regions of the left hemisphere. Tam *et al.* [6] developed a tablet based on touchscreen technology that was fMRI-compatible and also used it for the Trail Making Test, finding left hemisphere frontal lobe activations similar to the major results of Zakzanis *et al.*

Dysgraphia is a disorder where the subject has a deficiency in the ability to write, primarily in terms of handwriting [7]. The ability of subjects to transcribe their thoughts can be studied by monitoring their writing as they respond to stimuli during fMRI scanning. For many studies of dyslexia/dysgraphia, fine resolution is less centrally important than the ability to qualitatively grade responses and correlate them with brain activation, and a very simple, inexpensive system suffices.

Herein, such a system using a color map for pen localization is described, allowing the recording of written strokes as they occur in real time. Written responses by subjects can be monitored, and spelling and legibility assessed. Proof-of-principle responses and fMRI data are provided.

## 2. Experimental Section

### 2.1. Hardware

**Figure 1.**
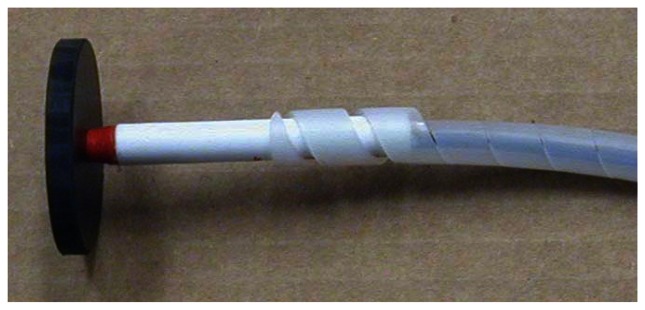
The pen assembly shown prior to securing with glue and enclosure in heat-shrink tubing.

Light is launched into, and collected from, a pair of 2 mm, multimode, plastic optical fibers. The fibers are epoxied in place within the emptied plastic shell of a disposable ball-point pen, in turn epoxied perpendicularly into the drilled-out center of a 2” diameter, 1/4” thick, acrylic plate. The ends of the fibers are ∼2 mm recessed from flush with the plate bottom, with one illuminating the contacted surface, and the other collecting the scattered light. The “pen” is shown in Figure 1.

**Figure 2.**
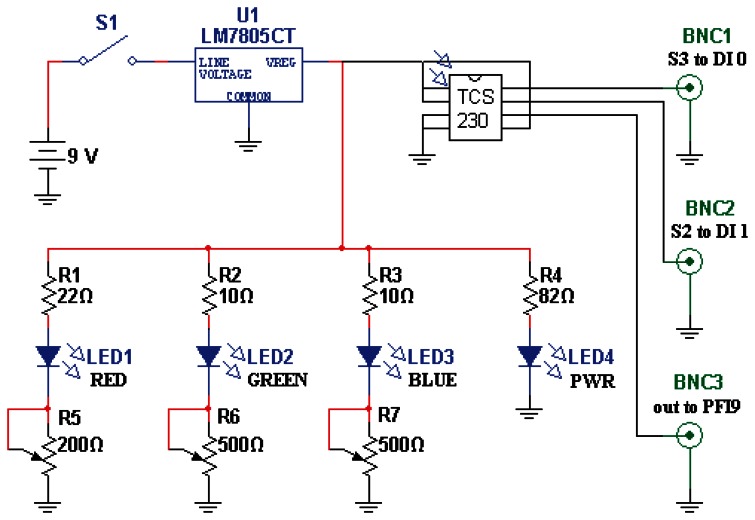
Computer-pen interface circuit diagram. DI0, DI1, and PFI9 are digital inputs of the PCI-6025E.

The color sensor is a TAOS TCS230 programmable color light-to-frequency converter (AMS-TAOS USA INC., Plano, TX, USA; Figure 2). Light from the three closely-adjoined red, green, and blue LEDs composing the “white” light source LED (a Luxeon Rebel “Neutral White” Star LED; Philips, Amsterdam, Netherlands) is contact-coupled to the map-illumination fiber, while the scattered-light-collecting fiber is abutted to the light-sensitive region of the TAOS sensor. Resulting pulse trains from the sensor are collected by a counter on a National Instruments NI PCI-6025E board (National Instruments Corp., Austin, TX, USA) and read into custom LabVIEW 2011 software (National Instruments Corp., Austin, TX, USA).

**Figure 3.**
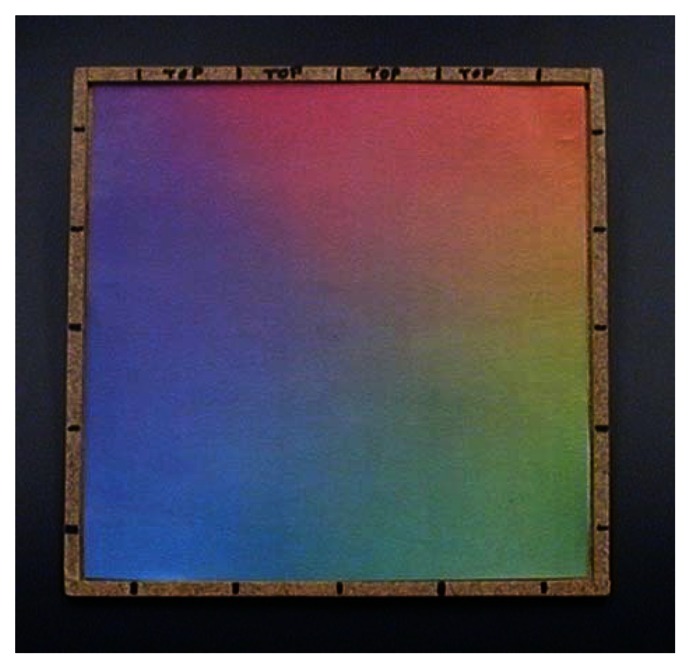
The color map.

The surface “written” upon is a continuously varying color map (Figure 3) printed from a Xerox WorkCentre 7428 printer (Xerox Corp., Norwalk, CT, USA) using conventional printer paper. In characterizing system response to isolated color gradients, we found saturation of the paper with pigment problematic; once saturated, critical gradient information is lost. We therefore found it advantageous to base our map on the printer's native pigments (cyan, magenta, and yellow) to reduce total pigmentation, and to spatially separate regions of maximal pigmentation using three overlapping linear gradients, one for each printer pigment, oriented at 120 degrees to each other. Subtle deviations from “white” (a 24-bit RGB value of 0xFFFFFF) were also difficult to detect, so only the intervening subsets of the full 0x00-0xFF range of each color gradient showing maximum sensitivity of color channel signal to incrementing RGB values were used to produce the final color map.

As overall luminance was expected to be sensitive to myriad artifacts such as fiber bending/misalignment, power supply variation, and color map wear, each of the sensor's red, green, and blue outputs are divided by their sum before use in determining position. The system was found to respond only weakly to even the brightest stray room illumination, such that no additional measures needed to be taken to exclude room lighting, and a sudden, precipitous drop in signal from the pen (>50% signal change in <250 ms) could reliably be interpreted as loss of contact between the pen and color map.

The map is held in a simple square hardboard frame 20 cm on a side, labeled with marks to indicate the grid of positions sampled during calibration, and with the top edge clearly indicated so as to maintain orientation between calibration and data collection. A plastic or glass overlay would surely serve to increase the resistance of printouts to wear. To date, however, we have preferred to simply print a fresh color map daily, eliminating concerns of signal offsets due to specular reflection.

### 2.2. Software

The software suite as currently implemented consists of four programs. The first simply queries the count total of each color channel of the sensor in turn, and displays these raw outputs as a scrolling chart to verify normal operation of the system and correct wiring of connections to the computer (crossed wiring permutes the color channels), and/or to characterize system performance when using new components/printer inks. The second guides the user through a calibration, prompting the user to place the pen at specified locations on the color map. A 5 × 5 grid of points was found to represent a favorable compromise between exhaustive sampling of the map and making recalibration fast and easy to perform and process. The color readings at this grid of points are saved in a file for subsequent reference.

The third program interpolates these calibration points with 2nd-order polynomial fits to each color channel independently, first along one axis, then along the other, producing an RGB value for each possible X-Y position. What is needed to interpret incoming pen data, however, is the inverse of this function, *i.e.*, an X-Y position for each RGB value measured. Due to noise/imperfection/wear of the color map, colors measured can vary somewhat from the values of the interpolated calibration. The subset of RGB values for which X-Y positions have been assigned—approximately a planar cut through RGB space—is thus “fleshed out” by assigning to each nearby-but-*unassigned* RGB value the X-Y position of the nearest *assigned* RGB value, essentially projecting the planar cut's X-Y assignments perpendicularly out onto neighboring planes in RGB space. Thus color values near to, but not falling exactly upon the interpolated calibration grid are accepted, and assigned the most probable position.

Still, this “fleshing out” is intentionally incomplete; the “projection depth” is limited such that not every point of the RGB color space is assigned an X-Y position, only those occurring near the slice through that color space that corresponds to the color map. Detection of an unassigned color can thus be used redundantly, in conjunction with an overall luminance drop, to detect removal of the pen from the map, either between strokes of a single letter, or upon completion of response to a stimulus. There is a trade-off however between strict rejection of off-map colors for robust lift-off detection and acceptance of such colors in the interest of noise tolerance. We found a tolerance of approximately ±5% of the R, G, and B byte values independently to be a favorable balance.

**Figure 4.**
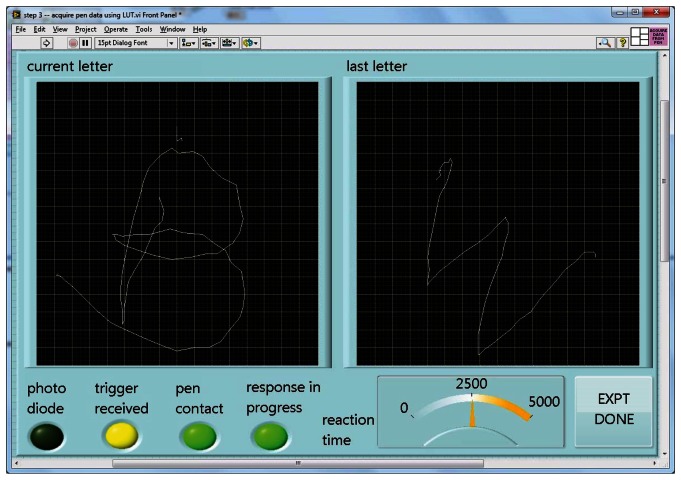
Run time display.

When run, this program turns a specified file of calibration readings and a desired number of pixels per axis into a lookup table of X-Y positions for each such RGB value, again saved to a file. Given such a table, the fourth and last program can accept and display incoming data as a character being drawn on the screen in real time (Figure 4).

As the software records the raw stream of time-stamped point coordinates (∼30/s) as well as saved images of each completed letter, dynamic aspects of the handwriting are captured for later analysis, such as stroke order, speed, hesitations, and lift-offs.

Both source code and executable software for generation of the color map, initial pen characterization, guided calibration, lookup table generation, and data acquisition are available at http://staff.washington.edu/freitz/MRIpad or upon request.

### 2.3. MRI Acquisition

Structural MRI and functional MRI scans were performed using a Philips 3T Achieva scanner (revision 3.2; Philips, Amsterdam, Netherlands) with a 32 channel head coil. Images were skull-stripped using the University of Oxford's *Functional MRI of the Brain* (FMRIB) Analysis group's *FMRIB Software Library (FSL)* [8,9], specifically the *Brain Extraction Tool* (BET) [10], and co-registered using *FMRIB's Linear Image Registration Tool* (FLIRT) [11,12].

Structural scanning was performed using the following parameters: Magnetization Prepared Rapid Acquisition Gradient Echo (MPRAGE) pulse sequence, TR/TE 7.6/3.5 milliseconds, acquisition matrix 256 × 256, acquisition voxel size 1 × 1 × 1 mm, reconstructed voxel size 1 × 1 × 1 mm, field of view 256 × 256 × 176 mm, TI delay 917 milliseconds, Sensitivity encoding (SENSE) factor 2 in the AP direction, slice thickness 1mm, number of slices 176, total scan duration 05:33.4.

The structural image was also registered to a standard 1 mm resolution MNI brain template (MNI152_T1_1 mm_brain.nii.gz) [13–15]. The FLIRT command provided transformation matrices that were used to transform a seed from Broca's area (MNI coordinates: −53, 20, 11 mm) to the individual functional image space using FSL's std2imgcoord command.

Functional scanning was performed using the following parameters: echoplanar gradient echo 2D pulse sequence, TR/TE 2000/25 milliseconds, acquisition matrix 80 × 77, acquisition voxel size 3 × 3 × 3 mm, reconstructed voxel size 3 × 3 × 3 mm, Field of view 240 × 240 × 99 mm, EPI factor 37, slice thickness 3 mm, number of slices 33, gap 0 mm, SENSE factor 2.3 in the AP direction, total scan duration 13:08.0, 387 dynamic scans, dummy scans 5.

Cubic regions of interest (ROI) of 3 × 3 × 3 voxels were centered over the Broca's seed voxel and the spatial mean over these 27 voxels was calculated at each TR, providing a seed time-series of average Blood Oxygen Level Dependent (BOLD) activity within the ROI. A 3 × 3 × 3 voxel target ROI was positioned throughout the brain and at each location the spatial mean of the target ROI was calculated. The corresponding target ROI time-series was correlated with the seed time-series to provide a Broca seed correlation map of the whole brain. Correlations were clamped at 0.999 and converted to z-scores.

Physiological monitoring was performed using the pulse oximeter heart signal and the respiration signal from the Philips physiological monitoring system.

### 2.4. Experimental Protocol

**Figure 5.**
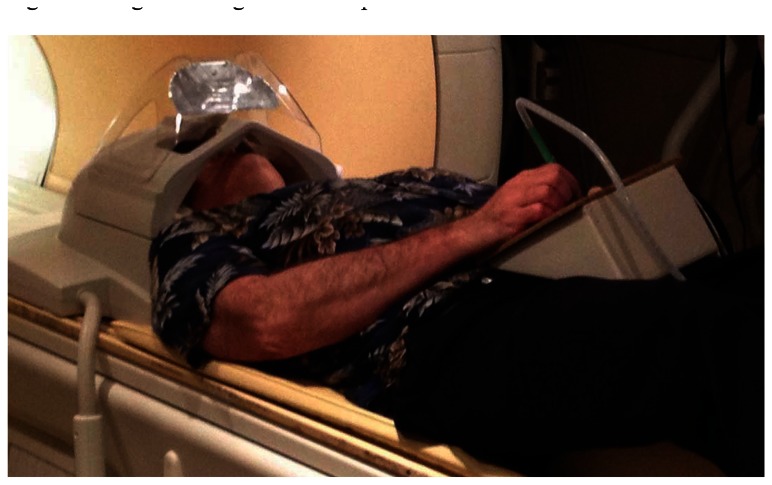
Tablet in use near the bore of the MRI scanner. The pen is connected to a fiber optic cable that goes through a waveguide that is positioned in the wall of the scanner room.

As implemented for lexical tasks in fMRI experiments, a photometer was placed in the corner of the display of a stimulus computer running E-Prime(R), and the LabVIEW software running on a separate, data collection computer waited for this photometer signal to trigger the start of a trial. The computers were linked by a serial cable. The subject was shown only the display from the stimulus computer via projection onto a mirror immediately above the head coil, while the experimenter observed the collection of real-time pen strokes on the LabVIEW runtime display (Figure 4). The subject wrote with the “top” of the pad toward their feet, and we found that inclining the pad on a 30 degree wedge of foam made this position quite comfortable (Figure 5). When the subject lifted the pen for a previously-specified period of time, the response was considered complete, the virtual writing area was saved in JPG format, and a serial signal was sent to the stimulus computer, cueing it to display the next stimulus. At the start of the next trial, the letter was transferred to a “last letter” display area, and the virtual writing area was again cleared for new input. Progress through the task was self-paced, with advancement automatically triggered by completion of subject responses.

We tested the fMRI-compatible pen with two 11 year-old subjects while they were performing writing tasks in a 3T scanner. Each task was presented in two 4-minute blocks to be used in an fMRI connectivity analysis preceded by a 6 second reminder instruction screen. Stimuli within each block were pseudo-randomly selected.

Before starting the tasks, subjects were given the following instructions: “In this part of the experiment, you will read letters and words on the screen and write with the pen. You have practiced these games before. Instructions will appear to remind you what to do next. Tell the experimenter when you are ready to start.”

The subject performed two tasks. Instructions for the first task were: “Write the letter that comes after the letter you see on the monitor. For example if you see a “b” write a “c”. Instructions for the second were “Write a letter to fill in the blank to make a real, correctly spelled word. For example if you see “_us” write the letter “b” to make the word “bus”.

## 3. Results and Discussion

The precision of the system was evaluated by moving the pen along a straightedge across the map at varying positions. As the disc on the end of the pen keeps the pen within the central 14 cm of the color map, at 32 pixels per axis, this translates to ∼4.5 mm per pixel. Along lines of constant X or Y, in the absence of averaging, calculated pixel positions showed a sub-pixel variation (standard deviation of ∼2.7 mm) uniformly over the center-most 50% of each range field, increasing to ∼5.4 mm over the outer-most quadrants of each axis. Time lag between pen motion and display of the stroke on the screen is ∼130 ms at present.

To assess whether the writing task using the pen induces head motion, the FSL software tool MCFLIRT [12] was used to assess head motion as a mean absolute displacement and as a relative displacement using the fMRI head signal. “Two subjects” motion was assessed while they performed a reading task (13 min) and a writing task using our pen (13 min) inside the scanner. Our preliminary data shows little difference between the motion during the reading task compared with the writing task, suggesting that use of the device does not necessarily increase head motion materially (Table 1).

**Figure 6.**
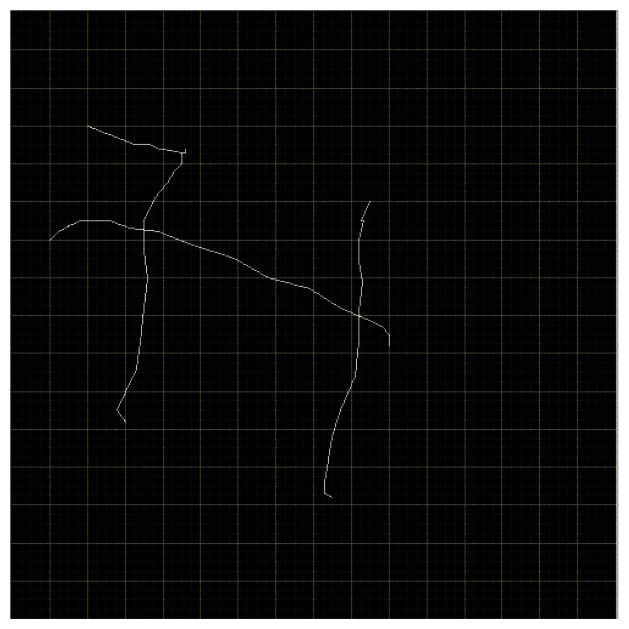
Subject response of letter “H”.

Figure 6 shows an example of output from the writing pen while the subject wrote the letter “H”. The letter is legible, though less precise than a corresponding pencil-handwritten letter on paper outside of the MRI. Letter quality is influenced by a number of factors apart from simple noise inherent to the electrical components used. One, while an interpolated calibration scheme saves time, it is imperfect, and will distort drawn lines slightly. Two, lines drawn are composed of segments bridging a finite set of points in a discrete grid, creating a slightly blocky effect. A rolling average of a modest number of points (five, in the case of Figure 6) greatly ameliorates this issue. Finally, the subject is lying down with the pad resting on their front while writing over-sized letters, a method with which subjects are unpracticed.

Still, even with low-cost components assembled by hand with only coarse precision, quite legible letters were obtained and displayed, and subject hesitations and overall performance were evident in real time.

**Figure 7.**
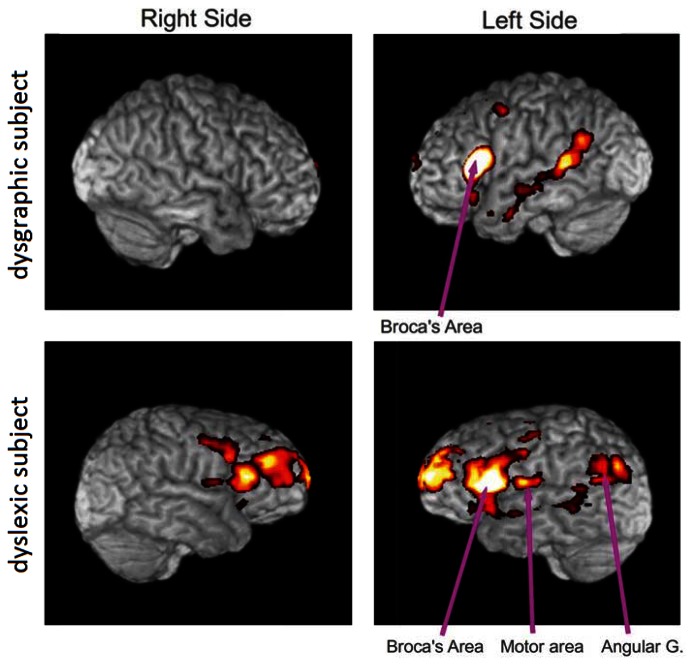
Dysgraphic and dyslexic subjects.

Functional MRI connectivity maps were obtained from a subject with dysgraphia and a subject with dyslexia (Figure 7). Both participants had completed a four-hour battery of tests of verbal intelligence quotient, spelling, word reading, word decoding, reading comprehension, and other writing tests prior to participation that carefully diagnosed them as having dysgraphia, dyslexia, oral and written language learning disability (OWL LD), or typical oral and written language learning development. These diagnostic decisions were based both on multiple normed measures and developmental, medical, and educational history, and rating scales completed by parents, using evidence-based criteria based on over two decades of NIH-funded programmatic research on normal language development and specific learning disabilities affecting oral and written language [16].

The functional connectivity maps were created from the fMRI scan while the subject was performing the writing task using the pen described herein. This connectivity analysis was based on a seed region in Broca's area on the left side of the brain. The clusters of activity are mainly in Broca's area, motor area, angular gyrus and frontal areas. Our preliminary results show more connections from Broca's area in the dyslexic subject than in the dysgraphic subject, but more data is required to establish statistical significance (studies in progress).

## 4. Conclusions/Outlook

In sum, we find the device as implemented usable for fMRI studies of dyslexia and dysgraphia. Construction was straightforward and inexpensive (∼$100 of parts beyond the cost of the LabVIEW development environment and data acquisition card). Use of the device was comfortable, did not induce prohibitive motion artifact during fMRI, and achieved high enough spatial (several mm) and temporal (∼30 points per second with ∼130 ms lag) resolution to legibly discern dyslexic/dysgraphic phenomena.

## Figures and Tables

**Table 1.** Reading *vs.* writing motion.

**Subject**	**Task**	**Mean Absolute Displacement (mm)**	**Relative Displacement (mm)**
1	reading	0.45	0.07
	writing	0.47	0.08
2	reading	0.19	0.06
	writing	0.22	0.06
